# Adaptive Response, Evidence of Cross-Resistance and Its Potential Clinical Use

**DOI:** 10.3390/ijms130910771

**Published:** 2012-08-29

**Authors:** Irina Milisav, Borut Poljsak, Dušan Šuput

**Affiliations:** 1 Institute of Pathophysiology, Faculty of Medicine, University of Ljubljana, Zaloska 4, Ljubljana SI-1000, Slovenia; E-Mail: dusan.suput@mf.uni-lj.si; 2 Faculty of Health Sciences, University of Ljubljana, Zdravstvena pot 5, Ljubljana SI-1000, Slovenia; E-Mail: borut.poljsak@zf.uni-lj.si

**Keywords:** oxidative stress, stress, adaptation, adaptive response, preconditioning, pretreatment, hormesis, autophagy, apoptosis, anti-apoptosis

## Abstract

Organisms and their cells are constantly exposed to environmental fluctuations. Among them are stressors, which can induce macromolecular damage that exceeds a set threshold, independent of the underlying cause. Stress responses are mechanisms used by organisms to adapt to and overcome stress stimuli. Different stressors or different intensities of stress trigger different cellular responses, namely induce cell repair mechanisms, induce cell responses that result in temporary adaptation to some stressors, induce autophagy or trigger cell death. Studies have reported life-prolonging effects of a wide variety of so-called stressors, such as oxidants, heat shock, some phytochemicals, ischemia, exercise and dietary energy restriction, hypergravity, *etc*. These stress responses, which result in enhanced defense and repair and even cross-resistance against multiple stressors, may have clinical use and will be discussed, while the emphasis will be on the effects/cross-effects of oxidants.

## 1. Introduction

Stress is any physical, chemical, or biological factor that adversely affects cellular physiology, growth or survival. Stress response is a mechanism used by organisms to adapt to and to overcome a stress stimulus. Different stressors trigger different cellular responses, namely induce cell repair mechanisms, temporary adaptation to some stressors, induce autophagy or trigger cell death. Redox regulation is an important mechanism for the regulation of cellular processes. There is a delicate balance between the production of reactive oxygen species (ROS) and the antioxidant defense. Having too many ROS in relation to the available antioxidants is often considered as a state of oxidative stress [1]. Oxidative stress can thus result from diminished antioxidants, like mutations decreasing the levels of glutathione (GSH) or manganese superoxide dismutase (MnSOD), or increased production of ROS, e.g., by exposure to toxins, due to mitochondrial damage or in chronic inflammatory diseases. Like in the case of other stressors, oxidative stress can result in various responses from increased proliferation [1], adaptation, cell injury and cell death, or a combination of these [2,3]. The stress response depends on the type of stressor and on its severity. For example, mild oxidative stress increases proliferation, moderate oxidative stress alters cell physiology to increase the level of protective systems that render the cell more resistant to subsequent insults. The mechanisms of stress responses include the activation of transcription factors and cell cycle arrest to allow repair of DNA damage. Severe oxidative stress results in severe oxidative damage and cell injury, cell senescence and/or cell death [2–4]. Here we shall focus on the adaptive responses.

## 2. The Molecular Mechanisms of Stress-Induced Adaptive Responses

The mechanisms of adaptive stress responses are largely unknown. Milisav and coworkers discovered that adaptation to stress involves the inhibition of apoptosis triggering through the intrinsic pathway in primary hepatocytes [5,6]. The main apoptosis initiator, caspase-9 is translocated from the cytoplasm to the nucleus as the consequence of moderate stress. Caspase-9 can be activated only after the activation of the main apoptosis execution caspase, caspase-3, in the stress-adapted cells [5]. This is in contrast to the situation in the unstressed cells, in which apoptosis is triggered by activation of the caspase-9 first. In other words, at least one of the main apoptotic triggering pathways is inactivated as the consequence of adaptation to stress. Nevertheless, apoptosis can be triggered even then by a more intense trigger than it is needed for the same effect in normal cells.

Cellular adaptations to stress can evade apoptosis by activating the so-called anti-apoptotic genes and proteins, which can temporarily block cell death [2,3,7]. The workable definition of the anti-apoptotic gene is that its product decreases or increases cell survival in response to a given dose of stress if its levels are decreased or increased (reviewed in [2]). The list of such genes and proteins is huge already and is expanding. Examples include anti-apoptotic proteins, FLICE-inhibitory proteins (FLIPs), B cell lymphoma-2 family members (BCL2), Inhibitors of apoptosis proteins (IAPs), ROS scavengers, like peroxiredoxin, glutaredoxins, superoxide dismutase, glutathione peroxidase, the ceramide utilizing Sphingomyelin Synthase 1 (SMS1), sphingomyelinase, *etc*. For more detailed information please refer to excellent reviews on the topics [2,3,7–10].

The exposure to stress releases intracellular second messengers, including ROS, calcium, 2′-Deoxyuridine 5′-Triphosphate (dUTP) and a sphingolipid ceramide [2,3,9–11], as well as currently unknown extracellular factors that enable stress adaptations at sites distant to the site experiencing moderate stress. This is seen in remote ischemic conditioning, where the limb ischemia induced by inflation of a blood pressure cuff can reduce myocardial ischemic injury in animal models (Section 4) [12]. The above-mentioned intracellular messengers participate in the propagation of survival and death signals and are covered in several reviews [2,3,9–11,13].

**Figure 1:**
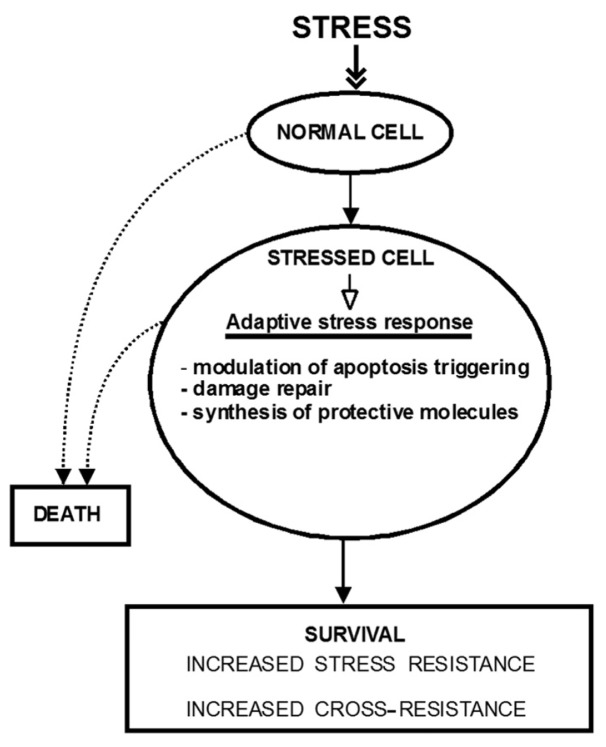
Adaptive stress response. Stress responses are mechanisms to adapt to and to overcome stress stimuli. Through them, the cells can restore stress-damaged structures or trigger cell death. Stress responses to mild/moderate stress may result in enhanced defense and repair and even cross-resistance to multiple stressors. This state is called adaptive stress response and it is achieved through multiple mechanisms, including modulation of apoptosis triggering [5], damage repair [14] and synthesis of protective molecules [2]. The solid line arrows point to the positive outcome, the dotted-line arrows to the negative one.

Many proteins involved in decisions of life and death are likely to play a part in adaptation to stress, since it is often the intensity and duration of the stress trigger that determines whether the cells can adapt to the stress or whether they will die as the result of it [3,14]. Also, any stressor will likely cause some damage to cellular structures, which will be removed and repaired. The cells may increase the synthesis of protective molecules, as is well established during oxidative stress (Figure 1).

### 2.1. Removal of Damaged Macromolecules

**Figure 2:**
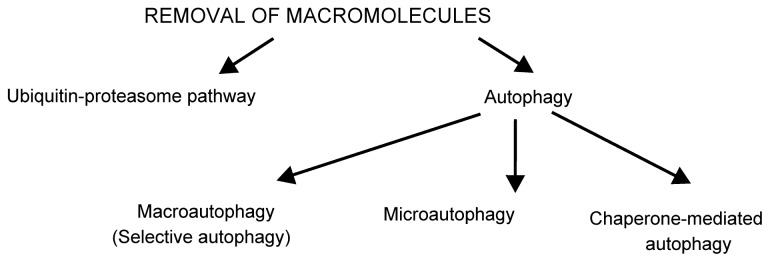
Pathways to remove stress-damaged macromolecules. The two major degradation pathways in eukaryotes are the ubiquitin-proteasome pathway (UPP) and autophagy. UPP is the degradation pathway for soluble proteins. In response to oxidative stress, it degrades oxidized proteins [15]. Through degradation of the transcription factor nuclear factor-E2-related factor 2 (Nrf2) that is involved in the transcriptional regulation of antioxidant enzymes, the UPP participates in the regulation of the intracellular redox status [41,42]. Autophagy is the name for several degradation pathways. Its best-described form is macroautophagy, the non-selective process of engulfment of cellular material into double membrane vesicles, which are delivered to lysosomes for degradation [43,44]. It has been recently appreciated that autophagy is also selective, called selective autophagy, in which the cargo is recognized by adaptor proteins [45]. Tiny portions of cytoplasm are sequestered and subsequently engulfed by lysosomes in microautophagy [46,47]. This process is well known in yeasts, however, there are not enough data on the mechanisms and physiological relevance of the mammalian microautophagy so far [46]. Chaperone-mediated autophagy (CMA), described only in mammals, delivers selected proteins into lysosomes through specific receptors [48].

There are two major degradation pathways in eukaryotic cells for removal of stress-damaged components: the ubiquitin-proteasome pathway (UPP) and autophagy (Figure 2). The UPP is the main pathway for degradation of soluble proteins, mainly in the cytosol. The UPP has been recognized recently to respond to oxidative stress and has a role in the degradation of oxidized proteins [15]. In the simplest form, UPP consists of labeling the protein target for degradation by covalent attachment of several molecules of a peptide ubiquitin and its degradation by cytoplasmic complex 26S proteasome. The 26S proteasome consists of a barrel-shaped catalytic core, also called 20S [16]. Two 19S regulatory particles (PA700) bind at either ends of the 20S cylinder and act as a gatekeeper for substrate entry. The ATPase subunits of PA700 contribute to unfolding of the protein substrates and delivery into the proteolytic chamber of 20S [17]. Other subunits of PA700 deubiquitinate the target proteins, or recruit polyubiquitinated substrates to the proteasome. Some proteins, including oxidized proteins, are degraded by the 20S proteasome in an ATP-independent and ubiquitin-independent manner [18–22]. Although many papers report the degradation of oxidized proteins in an ubiquitin-independent manner [18,20,23–29], there are also reports of degradation of ubiquitinated oxidized proteins [30–36]. Proteasome has an important role in selective degradation of oxidized proteins, however how the proteasome distinguishes oxidized proteins from the native ones remains to be determined. Sometimes the oxidation of specific residues results in changes in secondary, tertiary even quaternary structures of the proteins or even results in partial unfolding of the protein [28,37–40]. Such proteins may be recognized by molecular chaperones in a similar manner to other missfolded proteins [37]. The capacity of UPP to degrade proteins is altered by the cellular redox status. A mild to moderate oxidative stress promotes intracellular protein degradation by increasing susceptibility of proteins to degradation and enhancing the proteolytic capacity. Sustained oxidative stress inactivates proteasome without inhibiting the ubiquitination and results in accumulation of ubiquitin conjugates in the cells. Extensive oxidative stress inactivates the proteasome and inhibits the ubiquitination, resulting in accumulation of oxidatively damaged proteins [15].

UPP also has a role in the regulation of the intracellular redox status, as it degrades nuclear factor-E2-related factor 2 (Nrf2), which is a transcription factor involved in the transcriptional regulation of antioxidant enzymes [41,42]. Under normal cellular redox conditions, Nrf2 is ubiquitinated through binding the adapter protein Kelch-like ECH/associated protein (Keap1), which is an adaptor for one of the ubiquitin ligases (cullin 3); Nrf2 is subsequently degraded by a proteasome. When a cell is exposed to oxidative stress, Nrf2 dissociates from the Keap1/Cul3 complex, shifts into the nucleus and binds to an antioxidant response element (ARE) [49]. In this manner it regulates the ARE-mediated expression of antioxidant enzymes, like catalase, superoxide dismutase, NAD(P)H: quinone oxidoreductase, some glutathione *S*-transferases and γ-glutamate cysteine ligase regulatory subunit [50–52].

Autophagy, the other major degradation pathway, or rather pathways, degrades large cellular cargos that are firstly enclosed into double membrane vesicles; these vesicles are delivered to lysosomes for degradation. Almost any type of cellular material can be enclosed by a double membrane called phagophore, including whole organelles, protein complexes or aggregated proteins. The enclosed vesicles are called autophagosomes. These need to fuse with endosomes to become amphisomes [43] or with lysosomes to become autolysosomes [44]. Amphisomes can fuse sequentially with late endosomes or lysosomes. The process described above is macroautophagy, which is a non-selective process of engulfment. It is the best-studied form of autophagy and is often referred to as autophagy in the literature.

Another form of autophagy is selective autophagy with selective cargo recognition by adaptor proteins [45]. Ubiquitination, described above, has an important role in targeting the cargo for selective autophagy. The types of selective autophagy can be divided into three groups, those dependent on ubiquitin, on the autophagy Atg8 recognition motif, or on both [53]. Selective autophagy has a role in the case of large quantities of ubiquitinated proteins as in inclusion bodies and protein aggregates. The attachment of ubiquitins (polyubiquitination) is the most common to the lysine residue 48 (Lys^48^) of ubiquitin in UPP; however, the attachment to other lysine residues is also possible. Protein aggregates modified by ubiquitin chains linked to other lysines, including Lys^63^, Lys^27^, Lys^11^ are often recognized by selective autophagy. The autophagy adaptor proteins p62 and NBR1 (neighbour of BRCA1) recognize polyubiquitin chains [45] and expand the phagophore through Atg8. Protein p62 also regulates the stability of Nrf2 (see above) and nuclear factor κB pathway (NF-κB) [54]. Aggregates containing p62 are seen in neurodegenerative diseases, like Alzheimer’s, Parkinson’s and Huntington’s diseases, in myopathies and in liver diseases.

Macroautophagy also has an important role in the adaptation of organism to stress, like calorie restriction [55]. Oxidative stress is linked to mitochondrial dysfunction, as mitochondria generate and are targets for reactive oxygen species [56]. Defective mitochondria are degraded by autophagy, the activity of this pathway declines with age and in neurodegenerative diseases [57]. The cross talk between the redox signaling, mitochondrial dysfunction and autophagy is not well understood [58]. Autophagy is regulated by a protein complex mammalian target of rapamycin complex 1 (mTORC1). Protein mTOR (mammalian target of rapamycin) is a serine/threonine protein kinase and is the catalytic subunit of the complex mTORC1 [59]. mTOR inhibits autophagy by phosphorylating autophagy specific proteins Atg1 and Atg13; the phosphorylation inhibits the kinase activities of these autophagy proteins (Atg) and inhibits the autophagosome initiation [55].

The term microautophagy was proposed in 1966 when it was suggested that tiny portions of cytoplasm could be sequestered and subsequently engulfed by lysosomes [60]. Indeed, the lysosome-like organelles with multiple vesicles trapped in their lumens (multivesicular lysosomes) are often observed in mammalian cells; so are the lysosomes containing the organelles, like mitochondria or peroxisomes. Besides the images obtained by electron microscopy, other methods to detect microautophagy in mammalian cells are needed [46,47]. On the other hand, a lot of knowledge on microautophagy has been recently obtained from yeasts. Non-selective microautophagy is involved in the degradation of randomly sequestered portions of yeast cytosol. It starts by tubular invaginations of vacuolar membrane from which the small autophagic vesicles are released (pinched off). The selective forms of microautophagy are involved in the degradation of specific organelles, like mitochondria (micromitophagy) [61], parts of nuclei (piecemeal microautophagy) [62,63] and peroxisomes (micropexophagy) [64,65]. Yeast microautophagy is regulated by the target of rapamycin complex 1 (TORC1) [66], a conserved kinase complex, whose mammalian homologue is one of the key metabolic regulators (see below). As so little is known about the mechanisms and physiological relevance of mammalian microautophagy, there can be no definite judgement on the relevance of the results obtained in yeast for these processes in mammals as yet [46].

Chaperone-mediated autophagy (CMA) targets and delivers some proteins directly across the lysosomal membranes through a specific receptor [48]. This pathway has been described only in mammals. The basic machinery is composed of at least three types of proteins: (1) the chaperones that recognize the substrates, *i.e.*, the chaperone Hsc70 located in the cytosol and in the lumen of lysosomes [67,68]; (2) the receptors that bind and transport the substrates across the lysosomal membranes (Lgp96, human Lamp2a) [69]; and (3) the substrate proteins with specific motif KFERQ [70,71]. About 30% of cytosolic proteins carry this motif [71]. The signal transduction pathways involved in regulation may involve p38 mitogen-activated protein kinases (p38 MAPK) and are largely unknown [72]. CMA was initially identified as an inducible pathway in response to stress, there is, however, also a basal level of CMA activity [73]. The physiological roles of CMA include protein recycling and quality control, delivering antigens for antigen presentation by major histocompatibility complex class II, protein recycling in response to starvation and the removal of oxidized proteins during oxidative stress [48]. The activity of CMA increases progressively in the first 10 hours of starvation and, unlike macroautophagy, remains activated for the entire duration of starvation [48,74]. In this way, the essential proteins can be selectively maintained in the cytosol. CMA activation during nutrient deprivation is tissue- and celltype-specific and depends on age [74,75]. The upregulation of CMA was observed during oxidative stress *in vitro*; antioxidants could at least partly prevent the increased degradation of some proteins by CMA [75]. CMA declines with age, which may contribute to the accumulation of oxidized proteins characteristic in old organisms [76].

### 2.2. Damage Repair

**Figure 3:**
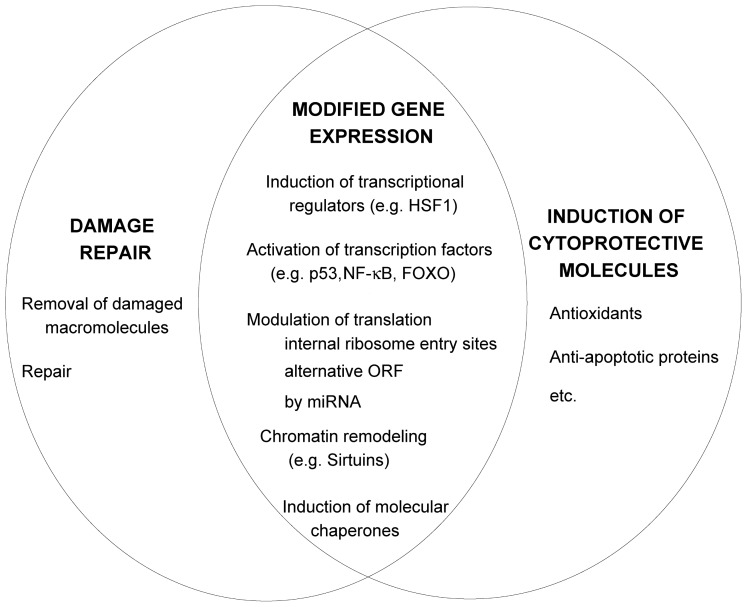
Cell repair and protection. Modulation of protein activity and expression has a key role in cell repair and protection during the adaptative stress response. The stress-modified regulation of transcription occurs, for example (1) by the induction of heat shock factors (HSF), which are the transcriptional regulators of genes encoding molecular chaperones and other stress proteins [80]; (2) by chromatin remodeling promoted by histone and DNA methyltransferases, demethylases, histone acetlytransferases and deacetylases [82]. The activity of histone deacetylases, like mammalian Sirtuin 1, is increased during nutrition stress [82,83]; (3) by the induction of various transcription factors [84–86]. The processes described above are interconnected at many levels; for example histone deacetylases sirtuins are involved in the regulation of transcription factors p53 and FOXO [87–90,91]. p53 has also non-transcripitonal activities; e.g., it interacts with apoptosis regulators [84]. The regulation of protein translation and activation is also crucial for stress-adaptation. The increased availability of a protein during the stress response can be achieved by reduced degradation (e.g., of p53), modifications in protein expression by translational regulation from the internal ribosome entry sites, upstream open reading frames, and modulation by micro RNA (miRNA) [14]. p53 and NF-κB regulate the transcription and processing of miRNA. The processes described are interconnected further; for example the different members of the heat shock protein family have a role in all of the described processes: in the activation of HSF, their transcription is stimulated during stress, they participate in repair processes and as anti-apoptotic proteins in combination with apoptosis regulators from the BCL2 family [3].

In addition to clearance of damaged macromolecules (described above), cellular repair is mediated through changed gene expression patterns [77], induction of molecular chaperones [78], growth arrest, *etc*. (Figure 3). The alterations of transcription during stress are often mediated by micro ribonucleic acids (miRNAs) (reviewed in [14]). These are short, noncoding, RNAs of about 22 nucleotides that bind to mRNAs and either accelerate their degradation or inhibit the translation of mRNA, *i.e.*, modulate the stability and/or translational potential of their targets. The stress responses modify the synthesis of miRNAs. The level of target gene repression depends on the relative concentrations of the genes inhibited by a miRNA and of a particular miRNA [79]. The outcome of miRNA repression depends also on the interactions with stress proteins that can modulate the activity of miRNA protein complexes, e.g., by inhibiting the access to target mRNA. The proteins transformation-related protein 53 (p53) and NF-κB (section 2.4) regulate the transcription and processing of miRNA. The subcellular location of miRNA can change as a consequence of stress. Most miRNA are diffused in the cytoplasm; they have to associate with the member of the Argonaute protein family for activity. After a maturation process at the Argonaute protein, mature miRNAs guide the Argonaute-containing complexes to target sites at mRNAs that are partially complementary to the miRNA sequence, and induce repression of gene expression at the level of mRNA stability or translation. miRNA are synthesized in about two hours, while the mature miRNA can peak in about 24 h. Therefore, the action of miRNA is delayed and can sometimes time the stress response. This is important in acute stress responses, such as during inflammation. Nutrient stress, temperature shock, DNA damage and hypoxia can lead to changes in gene expression by shutdown and reprogramming of protein synthesis through selective recruitment of ribosomes to mRNAs [77]. This is regulated by elements in untranslated regions of mRNAs, like internal ribosome entry segments, upstream open reading frames and miRNA target sites [14].

Heat shock factors (HSF) are inducible transcriptional regulators of genes encoding stress proteins, like molecular chaperones and others [14]. In vertebrates, HSF1 is the most important regulator of expression of heat shock proteins (Hsp) [80]. Hsp are molecular chaperones and among others assist proteins in folding or prevent and reverse protein missfolding and aggregation. In response to temperature shock or oxidative stress the HSF1 is converted from a monomer to a trimer. Monomeric HSF1 is a phosphorylated protein and interacts with Hsp90. Under stress, HSF1 dissociates from Hsp90, which enables its trimerization and binding to heat shock elements of Hsp genes. These processes are complex to enable a versatile regulation. HSF1 interacts with different Hsp at different phases of its activation cycle and is subjected to post-translational modifications, like phosphorylation, sumoylation and acetilation, many of them upon stress (reviewed in [80]). Both, HSF1 and HSF2 accumulate into nuclear stress bodies upon their stress-induced transcription. Nuclear stress bodies are thought to participate in rapid, transient, and global reprogramming of gene expression through different types of mechanisms including chromatin remodeling and trapping of transcription and splicing factors [81].

Molecular chaperones are proteins that assist the non-covalent folding or unfolding of proteins and the assembly or disassembly of multimeric macromolecular structures, but do not occur in these structures during their normal biological functions when these are in correctly folded states. Different classes of chaperones have different functions, including folding of newly made proteins, the repair of the damage caused by misfolding, membrane transport, keeping the protein precursor in translocation-competent state, assistance in protein degradation, *etc*. Chaperons function as intracellular quality control components for proteins. Many chaperons were discovered as their expression was elevated after heat shock, thus they were named heat shock proteins. Chaperons of the Hsp70 family interact with short extended peptide stretches of hydrophobic and basic amino acid residues of unfolded, natively folded or aggregated proteins [78]. The binding of Hsp70 prevents the aggregation of these proteins and can even induce conformational changes. Disaggregation of proteins is promoted by repeated Hsp70 binding and release to protein substrates. This is enhanced by cooperation with Hsp100 family chaperones. Substrate specificity of Hsp70, stabilization of Hsp70-substrate interactions and triggering of ATP hydrolysis by Hsp70 is promoted by several Hsp40 chaperones (J domain containing chaperones). Also, nucleotide exchange factors, like Hsp110 are required for ADP release and regeneration of ATP at Hsp70.

Chaperone Hsp90 is thought to bind and stabilize partially folded but inactive conformations of its substrates; some of these substrates may be recognized in extended conformations. Hsp90 hydrolyzes ATP like Hsp70; it interacts with a large number of co-chaperones. Its substrates are proteins involved in signal transduction, like protein kinases and transcription factors [78]. Co-chaperone Hop enables the cooperation of chaperones Hsp90 and Hsp70 by promoting their coupling.

TRiC (TCP-1 Ring Complex or chaperonin containing TCP-1; CCT) is an Hsp60 family member in the cytosol of eukaryotic cells. Like other Hsp60 family members, TRiC is a barrel-shaped protein complex that encapsulates substrates into a protected folding environment. Subunits of oligomers, with beta sheet secondary structures, are its substrates [78] and possibly late folding intermediates or misfolded proteins. Several small heat shock protein family members are located in the cytosol. ATP-independent small heat shock proteins (sHsp) bind misfolded proteins to prevent their aggregation and may loosen aggregates by coagreggation therefore facilitating subsequent re-folding by Hsp70. Aggregated proteins are presented to Hsp100 by Hsp70 or Hsp40. The solubilized protein can re-enter chaperone-mediated folding cycles or degradation by an ubiquitin-proteasome system. Irreversibly aggregated proteins are degraded primarily by selective autophagy (see above).

Proteins are often damaged as a consequence of stress in the course of their normal biogenesis, which is an error-prone process. For example, truncated polypeptides that result from incomplete translation, misfolded intermediates, and unassembled subunits of protein complexes have exposed hydrophobic regions, which may facilitate aggregation [14]. Neurodegenerative diseases may be the effect of cellular stress induced by defective clearance of aggregated proteins. Environmental stress triggers can induce nonnative posttraslational modifications and damage proteins in other ways and consequently induce cell stress as well. Even the normal metabolism in every cell is associated with some degree of protein damage. However, the extent of protein damage increases by adverse intrinsic and environmental conditions, like unbalanced protein synthesis, oxidative stress, metabolic stress, some environmental toxins and pollutants, elevated temperature, high-energy radiation, *etc*. To cope with a significant amount of protein damage, the damaged proteins are either repaired by molecular chaperones or degraded by the ubiquitin proteasome system or autophagy [78].

The stressed cells attempt to repair or degrade acutely damaged proteins by decreasing global protein translation and increasing the translation of molecular chaperones and proteins of the proteolytic system. The well-known adaptive responses in eukaryotic cells are the heat shock response (HSR) and unfolded protein response (UPR), a response to an accumulation of unfolded or misfolded proteins in the endoplasmic reticulum [78]. HSF1 modulates the HSR’s ability to up-regulate the expression of genes [80]. Under non-stress conditions, the monomeric HSF1 is in the cytosol bound by Hsp90, perhaps also Hsp70/40. These chaperones are tugged away under heat stress, which enables trimerization of HSF1, the activation of HSF1 trimer and expression of HSR target genes.

Chronic or unresolved ER stress can lead to apoptosis; therefore the cell’s ability to respond to stress in the endoplasmic reticulum (ER) is critical for its survival (reviewed in [78]). Several factors contribute to the development of ER stress including increases of protein synthesis or protein missfolding rates that exceed the capacity of chaperones, changes in calcium concentration in the ER lumen, oxidative stress and disturbances of the redox balance [92]. ER stress in eukaryotic cells is sensed by three upstream signaling proteins that initiate cascades of corrective reactions collectively known as the unfolded protein response. These are inositol-requiring protein-1 (IRE1), protein kinase RNA (PKR)-like ER kinase (PERK) and activating transcription factor-6 (ATF6). Prolonged activation of IRE1 can trigger apoptosis.

### 2.3. Increased Synthesis of Protective Molecules

**Figure 4:**
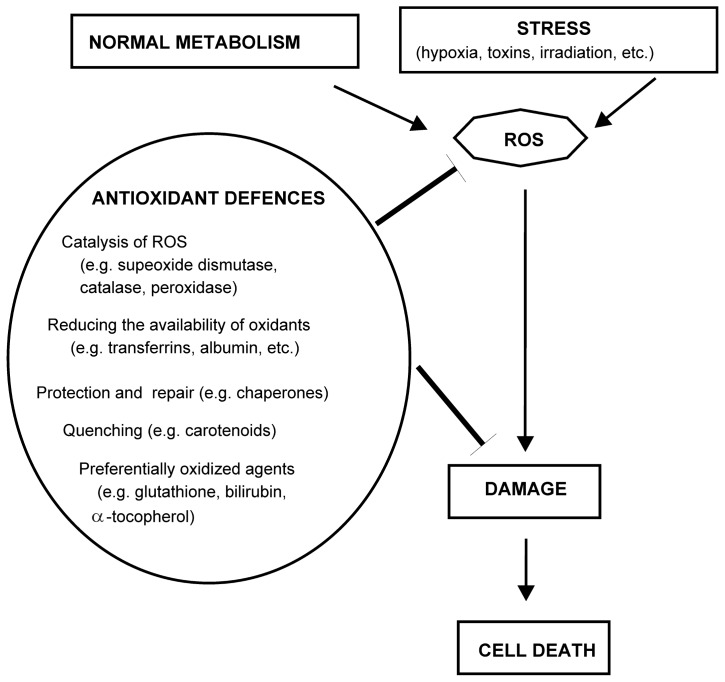
Antioxidant defenses. Antioxidant defenses can prevent the generation of reactive oxygen species (ROS) and intercept free radicals [95]. There are enzymatic defenses and non-enzymatic defense systems that protect the cells against free radicals and ROS [4]. The formation of some ROS is inevitable during normal metabolism and is necessary also for normal cell function.

The protective molecules include antioxidant enzymes, like superoxide dismutase (SOD), glutathione peroxidases, catalase, molecules like glutathione and the compounds derived from diet, like ascorbate, *etc*. (Figure 4). Regarding their function, the antioxidant defences can be divided into (1) enzymes that catalytically remove reactive oxygen species, like SOD, superoxide reductase, catalase and peroxidase; (2) agents that decrease the formation of reactive oxygen species by minimizing the availability of pro-oxidants, like iron and copper ions or heme. Such are transferrins, albumin, heme oxigenases, metallothionein, *etc.*; (3) proteins protecting biomolecules against oxidative damage, like chaperones; (4) physical quenchers, for example of a singlet oxygen by carotenoids; (5) agents that are preferentially oxidized, like glutathione, α-tocopherol, bilirubin, ascorbate, urate, albumin, *etc*. [93,94].

Since some free-radical production in cells is inevitable and can be very damaging, a defense system against the deleterious actions of free radicals has evolved. These are known as antioxidant defenses, and the two main categories are those whose role is to prevent the generation of ROS and those that intercept any radicals that are generated [95]. Beside enzymatic defenses, cells also possess a non-enzymatic defense system for the protection of cellular constituents against free radicals and reactive oxygen species (ROS) and for maintaining the cellular redox state. Glutathione (GSH) is the most abundant intracellular thiol-based antioxidant, prevalent in millimolar concentrations in living aerobic cells [93]. A second category of defence encompasses repair processes, which remove the damaged biomolecules before they accumulate to cause altered cell metabolism or viability [95]. Besides, natural antioxidants like vitamin C and E, carotenoids and polyphenols are generally considered to be beneficial components of fruits and vegetables. There are many more cytoprotective molecules in addition to the ones mentioned in this section, including anti-apoptotic proteins (Section 2) and others [2].

The BCL2 family of proteins consists of proapoptotic (Bax, Bak, Bid, *etc*.) and anti-apoptotic members (including Bcl-2, Bcl-*X_L_*, Mcl-1) [7,14,96]. Members of both groups are important regulators of apoptosis; the ratio of anti-apoptotic to proapoptotic BCL2 proteins regulates the cells’ sensitivity to apoptosis. Anti-apoptotic BCL2 proteins are important survival factors for many cancers and their overexpression has been associated with tumor initiation, progression, and resistance to current anticancer therapies [3,7,96–99]. Anti-apoptotic Bcl-2 is the best-studied member of the family. Among many other functions, it controls the integrity of the mitochondrial outer membrane in mammals, e.g., by inhibiting the pro-apoptotic Bax, it is involved in the regulation of autophagy by binding to Beclin-1 and has been implicated in the regulation of the intracellular redox status [96,100].

### 2.4. Stress Signaling Pathways

**Figure 5:**
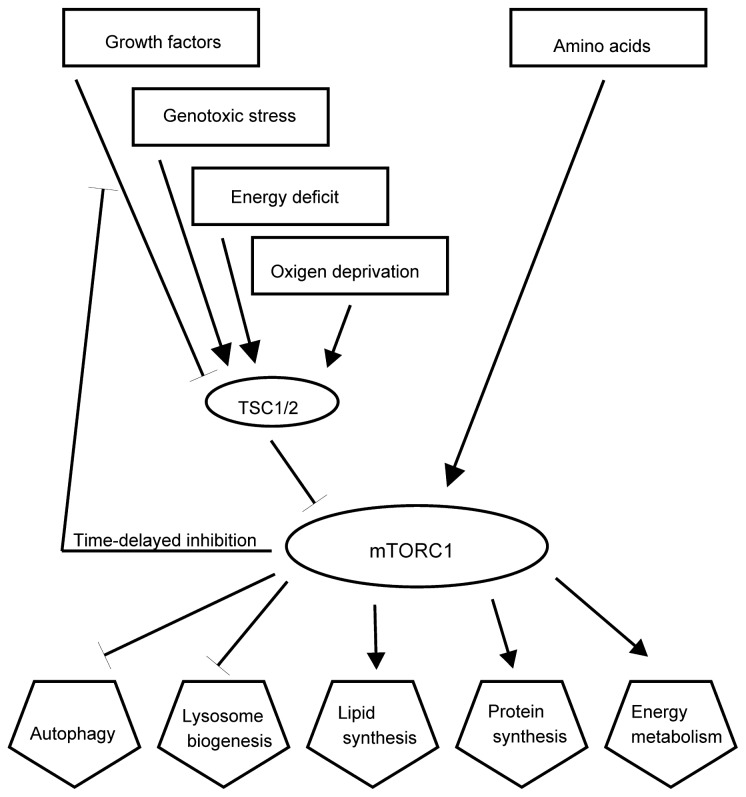
Simplified core stress signaling pathways. The kinase mTOR is a part of complexes mTORC1 and mTORC2. mTORC1 is at the bottom of the PI3-kinase (PI3K) and protein kinase B (AKT) signaling pathways, which receive signals from growth factor receptors at the plasma membrane [55]. mTORC1 also receives signals from p53 through AMPK in the case of DNA damage, through AMPK in the case of energy deficit, through IκB kinase β (IKKβ) during inflammation, *etc*. [104]. mTORC1 is inhibited by the heterodimer of tuberous sclerosis proteins TSC1 and TSC2 (TSC1/2). Sustained activation of mTORC1 blocks growth factor signaling through activation of the negative loop that inhibits PI3K signaling [101]. The activation of mTORC1 promotes protein synthesis mainly by phosphorylating the kinase S6K and through the regulator of translation 4E-BP1 [102,104]. The biogenesis of lipids is induced through the activation of transcription factors SREBP1 and PPARγ [103]. The energy metabolism is activated through the activation of expression of hypoxia inducing factor 1α (HIF1α) [104]. The active complex mTORC1 promotes anabolism and inhibits catabolism by blocking autophagy through phosphorylation of a complex that consists of ULK1-Atg13-FIP200 and inhibits lysosome biogenesis likely through the transcription factor EB (TFEB) [104]. mTORC2 (not shown) is activated by growth factors and regulates the organization of actin cytoskeleton through protein kinase C-α (PKC-α); it also regulates the serum- and glucocorticoid-induced kinase 1 (SGK1) that controls ion transport and growth; and AKT, which regulates metabolism, survival, apoptosis, growth and proliferation through phosphorylation of several effectors, including TSC1/2 [104].

As the consequences of stress are alterations in cellular metabolism, growth and division, it is no surprise that the main signaling pathways that regulate cell growth, metabolism, senescence and apoptosis also regulate the stress responses. The complex mTORC1 with its catalytic subunit mTOR regulates growth by maintaining the balance between the anabolic processes, like macromolecular synthesis, and catabolic processes, such as autophagy (Figure 5). mTORC1 mediates cellular responses to many types of stress, like DNA damage and drops in the level of energy, oxygen, amino acids and glucose. mTORC1 is at the bottom of PI3K and AKT signaling pathways, which receive signals from growth factor receptors at the plasma membrane and other inputs [55]. mTORC1 consists of mTOR and regulatory-associated protein of mTOR (raptor), mammalian lethal with Sec13 protein 8 (mLST8), proline-rich AKT substrate 40 kDa (PRAS40), and DEP-domain-containing mTOR-interacting protein (Deptor). The small G protein Rheb in the GTP-bound form stimulates the activity of mTORC1. Rheb is inhibited by the heterodimer of tuberous sclerosis proteins TSC1 and TSC2 (TSC1/2), which convert Rheb into an inactive GDP-bound state. Growth factors promote activities of several kinases, such as AKT, Erk and Rsk, which phosphorylate TSC1/2 and inhibit its activities, therefore enabling the mTOR signaling pathway. Sustained activation of mTORC1 blocks growth factor signaling through the activation of negative loops that inhibit PI3-kinase (PI3K) signaling [101]. The activation of mTORC1 promotes protein synthesis mainly by phosphorylating the kinase S6K and through the regulator of translation 4E-BP1 [102]. The biogenesis of lipids is induced through the activation of transcription factors SREBP1 and PPARγ [103]. The active complex mTORC1 promotes anabolism and inhibits catabolism by blocking autophagy through the phosphorylation of a complex that consists of ULK1-Atg13-FIP200 [104].

Stress, like hypoxia, energy deprivation, DNA damage and inflammation, activates TSC1/2, thus inhibiting mTORC1 activity. Amino-acid activation of mTORC1 is regulated by Rag GTPase and is independent of TSC1/2 [105]. A possible mechanism of mTOR regulation could involve the targeting of mTORC1 to different locations within the cells. The availability of amino acids stimulates the translocation of mTORC1 to the lysosomal surface, where it associates with Rheb resulting in activation of mTOR kinase [106,107]. In cells exposed to moderate starvation lysosomes relocate from close to the plasma membrane to the perinuclear microtubule organizing center [55]. During starvation, mTOR co-localizes with protein LC3 after the formation of autophagosomes [58]. This step may be sensitive to ROS, as the oxidation of mTOR results in the inhibition of its activity [108]. The association of mTORC1 with autophagosomes may facilitate autophagosome-lysosome fusion. The changes in lysosomal positioning in response to nutrients are mediated by intracellular pH, which is increased during starvation [55].

The changes in concentration of reactive oxygen species (ROS) and reactive nitrogen species (RNS) influence the regulators of autophagy [58]. Starvation induced autophagy is associated with increased oxidative stress in mammalian cells. Starvation probably triggers the accumulation of (hydrogen peroxide) H_2_O_2_, which was shown to be necessary for autophagosome formation in permanent cell cultures, mainly CHO cells [109]. The starvation induces formation of the complex of PI3 kinase and Beclin 1 with other signals and results in a local rise of H_2_O_2_ near mitochondria. This inactivates Atg4 and promotes lipidation of Atg8. Subsequently around the time of fusion with the lysosome, the vesicles are shifted to an H_2_O_2_ poor environment, where the active Atg4 can de-lipidate and recycle Atg8 [109].

H_2_O_2_ has a role in the response to nutrient starvation, rapamycin, TNF-α and nerve growth factor deprivation [110,111]. Both H_2_O_2_ and superoxide (O_2_^•−^) can induce autophagy [109,112]. Chen and coworkers found that the amount of autophagy induced by starvation, mitochondrial electron transport chain inhibition and exogenous H_2_O_2_ correlated with increased intracellular levels of O_2_^•−^ and decreased levels of H_2_O_2_ [112]. Cell death induced by starvation was increased by the inhibition of autophagy with autophagy inhibitors and superoxide scavengers and decreased by the autophagy by knocking down the expression of superoxide dismutase 2 (SOD2) [112]. The same authors reported the enhancement of autophagy induced by the inhibitors of mitochondrial electron chain transport with SOD2 inhibition that resulted in enhanced cell death. These data imply that autophagy is a cell survival mechanism that generates amino acids and fatty acids for growth or for the conversion into energy during starvation, however, if autophagy is prolonged it leads to cell death, since many proteins and organelles required for homeostasis and cell survival are degraded. The regulatory role of ROS on autophagy, its regulator and the regulator of homeostasis mTORC1 and other metabolic pathways is complex and more exciting discoveries will pave the way to improve the understanding of these processes.

p53 is a transcription factor, with transcription dependent and transcription independent activities [84]. It responds to many types of cellular stress, such as DNA damage, hypoxia and oncogene activation to regulate target genes that induce cell cycle arrest, apoptosis, senescence, DNA repair or changes in metabolism. p53 can interact with and modulate the activity of some members of the BCL2 family, the regulators of apoptosis [84]. Both, transcription dependent and transcription independent activities of p53 may lead to apoptosis; there are accounts when transcription independent mechanisms are essential for the full apoptotic response [84].

The concentration and activity of p53 are regulated mostly on the posttranslational level. The half-life of p53 protein in normal, unstressed cells is about six to 20 min, while it can last for hours under stress. Post-translational modifications are selectively activated by different stress signals. p53 is then modified by phosphorylation, methylation, monoubiquination, sumoylation, neddylation and glycosylation [14,85]. Different stress signals result in different post-translational modifications of p53 and transcription of different sets of genes.

In addition to the initiation of cell cycle arrest, senescence or apoptosis, p53 regulates cell growth and metabolism through down-regulation of IGF-1/AKT-1 and mTOR pathways. Insulin-like growth factor-1 (IGF-1) is secreted when insulin is secreted in response to high levels of nutrients. The binding of IGF-1 to a tyrosine kinase receptor recruits PI3 kinase and leads to its activation, which phosphorylates phosphoinositides, increasing their concentration at the plasma membrane [113]. Then lipid kinases are activated, like 3-phosphoinositide-dependent protein kinase 1 (PDK-1), which activates AKT by phosphorylation [114]. AKT is fully activated after the additional phosphorylation by mTORC2 [113]. AKT phosphorylates and inactivates proapoptotic protein BAD and activates antiapoptotic MDM2 [115]. Activated AKT translocates from the plasma membrane to the nucleus, phosphorylates the O subclass of the forkhead family of transcription factors (FOXO) [86], which leaves the nucleus. This results in transcriptional enhancement of proteins for oxidative phosphorylation, chaperones and the production of antioxidant proteins that decrease the levels of ROS [113]. The removal of FOXO from the nucleus turns off the production of p27, an inhibitor of cyclin D-cdk-4/6 kinases, activating the pathway for driving the cell into the cell cycle [113]. AKT also regulates mTORC1 by inhibiting TSC2, thus activating Rheb and mTORC1. mTOR regulates a translational control over cell growth, division and energy metabolism, whereas IGF-1/AKT regulate the transcriptional regulators of these processes. The result of the activated IGF/AKT pathway is therefore the promotion of growth and division and the inhibition of apoptosis.

Sirtuins are NAD^+^-dependent histone deacetylases whose activity can prolong the lifespan of organisms, such as yeast, worms and flies [116] and are important for aging regulation and caloric restriction-extended lifespan in *in vitro* cell models, animals and humans [82,83,117–121]. This is especially true for the mammalian sirtuin 1 (SIRT1) whose activity depends on NAD^+^/NADH ratio, which is the key indicator of oxygen consumption, thus SIRT1 may be responsive to the metabolic state of cells. SIRT1 increases stress resistance by negative regulation of p53 and FOXO [87–90]. It also initiates endocrine responses, including inhibition of adipogenesis and insulin secretion of pancreatic β cells by regulation of proliferator-activated receptor g coactivator 1α (PGC-1α) [122,123]. PGC-1α is also a regulator of mitochondrial biogenesis and function and is responsive to metabolic stress, therefore the number and capacity of mitochondria are adapted to the energetic demands of tissues [124].

Protein p53 also selectively regulates the transcription of genes that initiate cell-cycle arrest, DNA repair, senescence and apoptosis. It senses stress signals, like hypoxia, heat and cold shocks, DNA damage, nutrition starvation and oncogene activation [125,126]. In response to stress, p53 modulates IGF-1/AKT and mTOR pathways by the induction of expression of genes of negative regulators of these two pathways, like IGF-BP3 and PTEN, TSC2, AMPKβ1, Sestrin1 and Sestrin2 [126–129]. This results in the shutdown of cell growth and division and induction of autophagy. On the other hand mTORC1 phosphorylates and activates phosphatase PP2A, which dephosphorylates p53 at the site of its activation. Also, AMPK can directly and indirectly phosphorylate p53 in response to nutrient starvation [129]. More regulatory loops are known between these core pathways to balance the commitment to cell growth and division and stress responses. p53 responds to the intrinsic stress. Another transcription factor, NF-κB, responds to extrinsic stress [14,85]. NF-κB is a protein complex. In the absence of stress signals it is in the cytosol associated with the inhibitor IκB. IκB is phosphorylated upon stress by IκB kinase (IKK), then ubiquinated and degraded by a proteasome. The translocation of NF-κB to the nucleus enables the transcription of genes with NF-κB response elements. The activation of these genes results in cellular replication, inflammatory responses mediated by tumor necrosis factor, and cell survival signals. NF-κB also transcribes the gene of its regulator IκB-α resulting in a negative autoregulatory loop. The proteins p53 and NF-κB cannot function at the same time in the same cell; on activation of one, the other one is inactivated.

Ataxia-telangiectasia mutated (ATM) protein kinase has been recently recognized to have a role as redox sensor that controls the levels of reactive oxygen species in human cells [130]. The cells deficient in ATM have a reduced antioxidative response [131] and are more sensitive to treatment with oxidizing agents [132–134]. It is well established, that ATM is recruited to the sites of double strand DNA breaks and is among the kinases, which activate DNA repair and signaling pathways [130]. In addition, a novel mechanism was identified, where ATM was activated directly through oxidation. This was shown by studies *in vitro* in the presence of H_2_O_2_ [135]; oxidative stress may also inhibit ATM activation by DNA damage, as it disrupts the binding of the regulator proteins required for ATM activation through this pathway. Nevertheless, it is likely that ATM is exposed to the increase of ROS and DNA damage simultaneously, as ROS production also induces DNA damage. ATM interacts with all main signaling pathways mentioned above. ATM is required for NF-κB activation following exposure to ionizing radiation and reactive oxygen species [136]. ATM is linked to the expression of IGF1 receptor by an unknown mechanism, as there are reduced levels of this receptor in ATM-deficient cells [137,138]; the effects of ATM on insulin function and glucose metabolism may be mediated through phosphorylation of p53 [139]. The signaling between ATM and mTORC1 was identified where ATM phosphorylates LKB1 tumor suppressor proteins lead to the activation of AMPK in response to elevated levels of ROS [140]. ATM directly phosphorylates the subunit of hypoxia-inducible factor (HIF), HIF1α, which promotes the activity of TSC2, therefore inhibiting mTORC1 [141]. HIF is a protein complex that plays an integral role in the body’s response to low oxygen concentrations. The insight into the interconnections of the above-mentioned and other metabolic pathways are starting to shed light on the relationships between stress, longevity, control over metabolic networks and some disease pathways, and pave the way to many new discoveries needed to understand these core regulatory pathways.

## 3. Adaptive Response and Cross-Resistance

The adaptive response induced by low- or moderate-intensity stressor(s) could induce beneficial responses of an organism. Toxicologists sometimes use the term hormesis to refer to a biphasic dose response to an environmental agent that has a beneficial effect in low doses and a toxic one at high doses [142]. Therefore, hormesis can be defined as an adaptive response of cells and organisms to a moderate, usually intermittent, stress. Such stress inducers include oxidants, UV-radiation, heat shock, some phytochemicals, ischemia, exercise and dietary energy restriction as well as hypergravity. There are many reports of cross-resistance to the above-mentioned stressors in organisms from bacteria to humans; some examples will be mentioned below (Table 1).

### 3.1. Oxidative Stress Inducers (Oxidants)

Reactive oxygen species (ROS) are beneficial in moderate amounts and harmful in excess, leading to the state of oxidative stress. Human cells generate some hydrogen peroxide and other ROS molecules deliberately to use them as chemical signals to regulate glucose metabolism, cell growth and proliferation [172].

Adaptive responses after the exposure to sublethal levels of oxidants were observed in eukaryotic cells [143,144]. The protective responses to oxidants were observed upon the subsequent exposure to the same and related oxidants. Spitz and coworkers [143] observed resistance to normally lethal dose of H_2_O_2_ when CHO fibroblasts were preconditioned with the same agent. These cells were slightly resistant to heat shock damage as well. Also, pretreatment with heat shock somewhat protected the cells from the subsequent oxidative stress. Bovine vascular endothelial cells were also more resistant to oxidative damage after they had been pre-exposed to low levels of H_2_O_2_ [173]. Also, xanthine and xanthine oxidase pretreatment for one hour, which produces O_2_^•−^ and H_2_O_2_ resulted in resistance of CHO and rat hepatoma cells to subsequent H_2_O_2_ and γ-irradiation [144]. Even ten minutes after the pretreatment of CHO cells with H_2_O_2_ a cross-resistance to *N-*methyl*-N*′*-*nitro*-N-*nitrosoguanidine (MNNG) and γ-irradiation was reported; however, the cells were not cross-resistant to UV light [174]. The adaptive response induced by H_2_O_2_ was observed in human lymphocytes to protect these cells against otherwise lethal X-ray irradiation [145]. Some molecular changes were observed during the stress adaptation. In H_2_O_2_-pretreated bovine vascular endothelial cells there were increased activities of SOD, catalase and glutathione peroxidase [173]. Some increase in SOD was observed in xantine and xantine-oxidase treated cells; however, there was no increased SOD expression when the same cells were treated with H_2_O_2_ [144]. Also, the expression of the Hsp70 family of proteins was increased upon the pretreatment with xantine and xantine-oxidase [144], in pretreatment with H_2_O_2_ in chinese hamster fibroblasts [143], in reoxygenation of CHO cells after anoxia [175] and after the reperfusion injury in rat hearts [176].

The organisms can gradually adapt to a stressor. If adult rats are acclimated to elevated concentrations of O_2_, they can tolerate pure O_2_ longer than control rats, possibly due to increased synthesis of SOD in lungs, although a co-ordinated increase in antioxidant defences is essential for maximum protection [177]. For example, injection of liposomes containing catalase and SOD is more protective than SOD on its own. Like the rats, mice overexpressing MnSOD or CuSOD are more tolerant to hyperoxia [177]. Culturing HeLa cells at gradually increasing O_2_ concentrations over 21 months enabled the cells to grow at 80% O_2_; this was otherwise lethal [178].

### 3.2. Temperature Shock

The heat-induced adaptations were investigated by challenging cells and organisms with moderate stress that often resulted in anti-aging and life-prolonging effects [146]. It was demonstrated in a series of experimental studies that repeated moderate heat stress had anti-aging effects in human skin fibroblasts *in vitro*. The exposure to moderate heat stress could protect the cells from being damaged by oxidative stress or toxins such as cyanide [147]. In *C. elegans*, brief thermal stress was sufficient to induce thermotolerance and caused small but statistically significant increases in lifespan [148]. Overexpression of certain HSPs significantly extended the longevity of normal-lived animals [179,180]. HSP induced increase in glutathione (GSH) may offer protection against subsequent oxidative stress (e.g., induced by H_2_O_2_), since small Hsp protect against oxidative stress through a glucose-6-phosphate dehydrogenase (G6PD)-dependent ability to increase and uphold GSH in its reduced form [181]. The long-lived insulin/IGF-1 mutants of *C. elegans* are resistant to both oxidative and thermal stresses, possibly through increased expression of heat shock proteins and antioxidant enzymes [182].

Cold stress also increases resistance to other stresses, like heat and fungal infection in aged *Drosophila melanogaster* flies [149]. Hormetic effects of repeated exposures to cold at young age reflected on longevity, aging and resistance to heat or cold shocks in *Drosophila melanogaster* [150].

### 3.3. Exposure to Chemicals and Toxins

As it was mentioned above, the exposure of cells to moderate heat stress can protect them from being damaged by oxidative stress or toxins such as cyanide [147]. Similarly, when cells are exposed to a low dose of the mitochondrial uncoupling agent 2,4-dinitrophenol they are less vulnerable to ischemia [151]. Recent findings imply that health benefits of many phytochemicals may be the consequence of cross-resistance in which a phytochemical activates one or more adaptive stress response pathways [183].

Beneficial effects for health have been demonstrated for many chemicals isolated from plants [184,185]. Initially it was thought that the antioxidant activity of phytochemicals is responsible for their health benefits [186]. It still stands that the oxidative stress contributes to the pathogenesis of many diseases and to the ageing process itself [187,188], and that fruits and vegetables contain the substances that exhibit direct free radical-scavenging properties at high concentrations. Micromolar concentrations of vitamin E and numerous polyphenols can protect a variety of cells against oxidative stress in cell culture models of cancer, atherosclerosis and neurodegenerative disorders [189–191]. However, the clinical trials and primary prevention studies of high doses of antioxidants in humans have not fulfilled the expectations [192]. Low levels of ROS function as signaling molecules and may induce adaptive responses, therefore over-consumption of exogenous antioxidants can lead to “anti-oxidative” stress when antioxidants attenuate or block adaptive stress responses [193]. Through the consumption of fruits and vegetables we receive relatively low amounts of phytochemicals. At least some of them may induce adaptive responses through the mechanisms described in Chapter 2. This notion is supported, among others, by the studies of experimental models of cancer [194], cardiovascular disease [195] and neurodegenerative disorders [183]. Examples of the effect of phytochemicals on the activation of stress induced pathways include the activation of the transcription factor Nrf-2 and its target, the antioxidant response element (ARE) by sulforaphane and curcumin [196]; activation of histone deacetylases and their target FOXO transcription factors by resveratrol [91]; and the activation of the transient receptor potential calcium channels by capsaicin and allicin [197]. While these phytochemicals may deter microorganisms and insects from eating a particular plant, they are resorbed in relatively low doses by humans and may induce cellular stress response pathways and improve the survival of cells.

### 3.4. Ischemia

Preconditioning ischemia occurs when an organ, often the heart or brain, is exposed to a brief period of moderate ischemia. The cells subsequently become more resistant to ischemia at a full-blown heart attack or stroke [198,199]. A brief period of ischemia followed by reperfusion in pig hearts resulted in prolonged depression of contractile function. This effect was attenuated by antioxidants [200]. Repeated brief periods of ischemia/reperfusion led to a quicker return of contractile function on reperfusion; this adaptive response was not seen in the antioxidant treated animals. The question arises whether such an adaptive response could be induced by other triggers?

Environmental factors that may improve the performance of the cardiovascular system upon stress are caloric restriction and exercise. Both can induce oxidative and metabolic stresses and activate stress resistance pathways in cells of different organs [201]. In addition to ischemia, the protective response can be initiated by multiple other stressors including heat stress, exercise, adrenergic drugs and even noise [202]. Clinical application of subjecting patients and their organs to transient stresses may broaden the therapeutic potential for the future clinical settings.

### 3.5. Physical Activity

A single bout of exercise can induce oxidative damage in an untrained person [203], while moderate daily exercise appears to be beneficial; for example, it reduced the damage to rat skeletal muscle [153]. Endurance training adaptation causes increased efficiency in ATP synthesis at the expense of a potential increase in oxidative stress that is likely to be compensated by enhanced activities of antioxidant enzymes [204] and proteasome [205]. Increased O_2_ consumption during sporting activity also increases free radical defense systems for example by increasing muscle levels of (SOD), glutathione peroxidase and reduced glutathione (GSH) [206]. Regular or repeated bouts of exercise are associated with lower resting metabolic rate, higher antioxidant activity, and lower oxidation of LDLs and more protection against oxidation of proteins and DNA [207,208]. Radak and coworkers suggest that the beneficial effects of regular exercise are partly based on the ROS generating capability of exercise [209]. Single bouts of exercise increase, while regular exercise decreases the oxidative challenge to the body by regulating oxidative stress-induced adaptations such as induction of antioxidants, DNA repair and protein degrading enzymes.

Cross protection between physical activity and aging was also noticed. Exercise improves the average lifespan of rats given access to voluntary running wheels compared to their sedentary counterparts [155]. Taking up a moderate sports activity is associated with lower death rates from all causes among middle-aged and older men [158].

There are reports of cross protection between endurance training and lower susceptibility to ischemia-reperfusion insults in animals *in vitro* (e.g., isolated perfused model), *in situ* (e.g., open heart surgery model) or *in vivo* [154–157].

### 3.6. Caloric Restriction

Caloric restriction (CR) refers to limiting the dietary energy intake while maintaining all essential nutrients; it is the most effective procedure to increase the average and maximum life spans and lower the risk of age-related diseases in many organisms [164] (prokaryotes [160–162] and eukaryotes [163,164,210]). Carbohydrate-starved cultures of *Lactococcus lactis subsp. lactis* IL1403 had enhanced resistance to heat, ethanol, acid, osmotic, and oxidative stresses [162]. Starved *Aeromonas hydrophila* developed increased resistance to lower temperature and ethanol stresses [160]. Starvation induced cross protection against heat or H_2_O_2_ in *Escherichia coli* [161]. The examples of the CR studies on primates include a 20-year longitudinal adult onset CR study in rhesus monkeys, where the baseline intake of calories was reduced progressively by 10% per month and maintained reduced by 30% from unrestricted intake for the duration of the experiment. Such CR significantly reduced the incidence of diabetes, cancer cardiovascular disease and brain atrophy [163]. The effects of the CR on the human body were studied in a limited number of experiments (reviewed in [211]). Globally, the effects of CR are achieved through modulating the IGF1/insulin and mTOR signaling pathways, gene expression regulation, including through DNA methylation and histone acetylation/deacetylation.

### 3.7. Hypergravity

Hypergravity is defined as the condition when the force of gravity exceeds the gravity level on the surface of the earth. Hypergravity can be regarded as stress since the subjects exposed to high *g*-loads have to adapt to a higher weight, which increases the metabolic demand on the organism. The higher metabolic rate may increase the production of free radicals and oxidative stress, thus inducing a higher activity of the antioxidant systems. The beneficial effects of hypergravity on longevity and aging were observed in male flies of *Drosophyla* sp. that lived in hypergravity at a young age [150,165]. Chronic exposure to hypergravity increased the thermotolerance in young and middle-aged *Drosophila* [150]. The exposure to hypergravity can induce an adaptive response. This mechanism of adaptation does not seem to include the increased antioxidant response [165,212]. Interestingly, hypergravity did not increase the synthesis of heat shock proteins, although there was an increase in total protein synthesis after a heat shock when the model organisms were exposed to hypergravity at a young age [213]. To clarify these observations, the mechanisms of adaptation to hypergravity will need to be investigated further.

The challenge with hydrostatic pressure improved post-thaw survival of cryopreserved mouse blastocysts [166] and increased *in vitro* developmental competence of pig oocytes [167] and vitrified bovine embryos [168]. The pre-treated porcine oocytes led to the birth of healthy piglets [167]. High hydrostatic pressure pretreatment also improved the survival rates, motility and fertility of bull and boar semen [169,170]. Therefore, induction of an adaptive response by high hydrostatic pressure has been proven in animal reproductive cells and needs validation in clinical practice.

## 4. Clinical Applications of Adaptive Stress Response

Understanding the adaptive response is of clinical interest. There are many examples where the adaptive response to stress is beneficial, however, the stress adaptations are potentially harmful in the case of prolonged stress. It has recently been established that the presence of stress phenotypes is one of the hallmarks of cancer [214]. Prolonged stress is connected to development or deterioration of various pathologies: For example, the accumulation of unfolded proteins contributes to many neurodegenerative diseases [78]. It needs further investigation to find out whether the pathologies are due to lack of adaptive response, the adaptive response being not efficient enough or because of the prolongation of this process.

Procedures to induce the adaptive response are used in clinical practice already. Ischemic reperfusion injury can be decreased by ischemic preconditioning [215]. Since it has been described in heart [216] and in brain slices there have been numerous reports of ischemic preconditioning, mainly of heart, brain, kidney and liver (for a review consult [217–220]). Procedures with great clinical potential include post-conditioning and remote ischemic conditioning (Section 2). The principles of ischemic preconditioning are applied after the ischemic event in post-conditioning [12,221,222]. Clinical investigations of this procedure were initiated within two years of the first report of the post-conditioning to reduce the myocardial infarct size on animals in 2003 [12,223]. The results of these clinical trials are promising; the improvements of the early markers of infarct size and the myocardial function for up to one year after the infarction were reported. The protective roles of post-conditioning are being investigated also in other organs of model organisms, for example to reduce the brain damage [221].

Remote ischemic conditioning is of practical importance, as limb ischemia induced by inflation of a blood pressure cuff can reduce myocardial ischemic injury [12]. The cardioprotective role of this procedure was reported in various animal models, when ischemia was applied as remote preconditioning or remote post-conditioning. The meta-analysis of 17 randomized controlled trials on the effects of remote ischemic preconditioning on myocardial and renal injuries pointed out the heterogeneity of results and no relevant publication bias [224]. The authors concluded that remote ischemic preconditioning has potential beneficial effects on serological markers of myocardial and renal injury during cardiovascular interventions.

Modulation of stress mechanisms may improve the outcome of transplantations. For example, cold ischemia pretreatment correlates with increased regeneration of epithelial cells immediately after transplantation of kidney allografts [14,225]. The adaptations to increased hydrostatic pressure improve the survival of murine and bovine blastocysts after freezing or in suboptimal culture conditions [226]. On the other hand, the deterioration of mechanisms of cellular adaptation to stress results in reduced survival of kidney grafts. For example, the chances of successful kidney transplantation are reduced in old donors [225]. Namely, many processes associated with aging are general pathways involved in tissue damage and stress responses. Modulation of adaptive response may delay aging; the adaptive response triggered by 1% DMSO substantially extends the lifetime of primary hepatocytes in the culture [6,152].

Pretreatments of cells to manipulate the stress response pathways can reduce cellular damage and improve transplantation outcome. Preconditioning of model cells of retinal pigment epithelium with non-lethal oxidative stress protects these cells from cell death induced by oxidative-stress [227]. The application of moderate shear stress on liver tissue slices was better than no shear or high shear stress for the survival of cells. Therefore, perioperative flow management is needed to regulate shear stress, *i.e.*, to avoid the excessive shear stress on liver tissue upon a massive liver resection [171]. Like excessive shear stress, the absence of shear stress was also damaging for the tissue; it resulted in destruction of sinusoidal structures.

Targeting the expression of proteins involved in stress response increased the stress resistance of grafts. Examples are increased synthesis of heme oxygenase-1 that improved the viability of liver graft in Lewis rats, presumably by increasing the cellular resistance against oxidative injury [228]. Heat shock proteins were protective in models of transplantation; however, there is a need to develop strategies for their upregulation in clinical practice. Overexpression of Hsp90-binding agent geldanamycin and some of its analogs protected renal cells from oxidative stress and reduced kidney ischemia-reperfusion injury in a mouse model [229]. Rat mesenchymal stem cells overexpressing Hsp20 were resistant to oxidative stress and had about a twofold higher survival rate after transplantation into infarcted hearts [230]. The evidence that the adaptive response may improve the survival of grafts is important, as the cells are often transplanted into an environment that is damaging to the original tissue. Temporary adaptation to stressors may be crucial for the survival of grafted cells; this can be achieved by inducing the adaptive response.

## 5. Conclusions

Adaptive response is an adaptation of healthy cells to moderate environmental stress. It improves their chances of survival under stress conditions, *i.e.*, in the presence of the stressor that initially induced the stress response or another stressor; cross-resistance is achieved in the latter case. Manipulating the cellular mechanisms to adapt or cross-adapt to stress is important in clinical settings, for example, to improve the outcomes of damaging events, such as transient ischemias, for improvement of the success rate of transplantations and perhaps even of surgical procedures. The prolonged adaptation to stress with inefficient damage repair may lead to cancerogenesis, thus there is a need for manipulation of this as well. Finally, the cellular adaptation to stress is important to improve the quality of life, as the stress-adaptation mechanisms can increase the longevity and delay the onset of age-related diseases.

## Figures and Tables

**Table 1:** Examples of cross-resistance to stressors.

Stressor	Cell Type/Organism	Cross-Resistance	Reference
H_2_O_2_	CHO cells	Heat shock	[143]
		*N-*methyl*-N*′*-*nitro*-N-*nitrosoguanidine (MNNG)	[144]
		γ-ray irradiation	[144]
	Human lymphocytes	X-ray irradiation	[145]

Heat	CHO cells	H_2_O_2_	[143]
	Human skin fibroblasts	Delayed aging	[146]
	Renal epithelial cells	Cyanide	[147]
	*C. elegans*	Increased lifespan	[148]

Cold	*Drosophila melanogaster*	Heat stress	[149,150]
		Increased lifespan, delayed aging	[150]

Chemicals			
Xanthine and xanthine oxidase	CHO cells	H_2_O_2_ and γ-irradiation	[144]
	Rat hepatoma cells	H_2_O_2_ and γ-irradiation	[144]
2,4-dinitrophenol	Rat brain cells	Ischemia	[151]
Dimethyl sulfoxide	Hepatocytes	Improved survival	[152]

Exercise	Rat skeletal muscle	Oxidative stress	[153]
	Rat heart	Ischemia	[154]
	Rats	Delayed aging	[155]
		Ischemia	[156,157]
	Humans	Delayed aging	[158]
		Ischemia	[159]

Caloric restriction	*Aeromonas hydrophila*	Lowered temperature, sodium, and ethanol stresses	[160]
	*Escherichia coli*	Heat stress and H_2_O_2_	[161]
	*Lactococcus lactis* subsp. *Lactis*	Heat, ethanol, acid, osmotic, and oxidative stresses	[162]
	*Rhesus* monkeys	Delayed aging	[163]
	Humans	Delayed aging	[164]

Hypergravity	*Drosophyla* sp.	Thermotolerance	[150]
		Longevity, delayed aging	[150,165]

Hydrostatic pressure	Mouse blastocysts	Improved survival	[166]
	Pig oocytes	Improved survival	[167,168]
	Bull, boar spermatozoa	Improved semen quality	[169,170]

Shear forces	Liver tissue	Improved survival	[171]
